# Editorial: The underlying relationship between sleep and neurodegenerative diseases

**DOI:** 10.3389/fnins.2022.1117265

**Published:** 2023-01-05

**Authors:** Gerard Piñol-Ripoll, Marcelo M. S. Lima, Shi-Bin Li, Adriano D. S. Targa

**Affiliations:** ^1^Cognitive Disorders Unit, Clinical Neuroscience Research, Hospital Universitari Santa Maria, Biomedical Research Institute of Lleida (IRBLleida), Lleida, Spain; ^2^Department of Physiology, Federal University of Paraná, Curitiba, Brazil; ^3^Department of Pharmacology, Federal University of Paraná, Curitiba, Brazil; ^4^Department of Psychiatry and Behavioral Sciences, Wu Tsai Neurosciences Institute, Stanford University, Stanford, CA, United States; ^5^Department of Anesthesiology, Zhongshan Hospital, Institute for Translational Brain Research, State Key Laboratory of Medical Neurobiology, MOE Frontiers Center for Brain Science, Fudan University, Shanghai, China; ^6^Translational Research in Respiratory Medicine, Hospital Universitari Arnau de Vilanova-Santa Maria, Biomedical Research Institute of Lleida (IRBLleida), Lleida, Spain; ^7^CIBER of Respiratory Diseases, Institute of Health Carlos III, Madrid, Spain

**Keywords:** sleep, circadian rhythms, neurodegenerative diseases, Alzheimer's disease, Parkinson's disease

Alterations in sleep and circadian rhythms are prevalent features in several neurodegenerative diseases. Manifestations in this regard are diverse, varying according to the type of neurodegenerative disease, the stage in which the patient is in the course of neurodegeneration, and the pharmacological treatment, among others. Patients with Alzheimer's disease (AD) report difficulty in falling asleep, frequent night-time awakenings, and excessive daytime sleepiness (EDS), in concordance with alterations in the sleep architecture and sleep efficiency often observed through objective evaluations (Peter-Derex et al., 2015). Sleep-wake disturbances are the most common non-motor symptoms among Parkinson's disease (PD) patients, represented by similar manifestations as well as classic features such as rapid eye movement (REM) behavior disorder (RBD) and restless legs syndrome (RLS) (Stefani and Högl, 2020). The circadian alterations comprise a decrease in the amplitude of the rhythm among PD patients whereas individuals with AD present an increased fragmentation and shifts to a later time in the circadian phase (Leng et al., 2019).

Yin et al. presented an elegant review discussing the current evidence related to sleep disturbances in autoimmune neurological diseases, a new category of diseases associated with antibodies against neuronal cell surfaces or synaptic proteins. The authors described the sleep alterations associated with each type of autoimmune neurological disease, presented as EDS, RBD, sleep-disordered breathing (SDB), non-rapid eye movement sleep parasomnias, and narcolepsy. The review by Chapelle et al. gave additional insight into the relationship between epilepsy and dreaming. The authors reported the available evidence demonstrating that the content of dreams is altered among epilepsy patients, mainly related to familiar settings surrounded by a negative context in detriment of success and sexuality-related settings. Additionally, the frequency of dream recall is affected in those patients.

Sleep and circadian alterations often precede the diagnosis of neurodegenerative diseases. Insomnia, sleep fragmentation, and SDB are all independently associated with an increased risk of developing several neurodegenerative diseases (Shi et al., 2018). In terms of circadian alterations, studies suggest that reduced amplitude and higher fragmentation of the rhythms as well as circadian phase shifts precede the risk of AD while inactivity during the day precedes the risk of PD (Leng et al., 2019). Rabinowitz et al. provided an interesting report, revealing that more stable circadian rest-activity rhythm was related to slower cognitive decline among cognitively healthy adults. The authors also demonstrated that such relationship was pronounced among women, people of color, aged individuals, and apolipoprotein E e4 allele carriers. Similarly, Liu et al. discussed the circadian regulation of several factors that could increase the risk of stroke. The authors comprehensively addressed the variation in stroke severity and its adverse outcomes according to the time of day in which it occurs and the discrepancy in the pharmacological treatment efficacy depending on the time of day in which it is administered.

Studies focusing on the mechanisms by which sleep and circadian alterations could increase the risk of neurodegeneration and vice versa are paramount to disentangling this complex relationship. Neurodegeneration inherent to each disease affects relevant nuclei and pathways associated with sleep and circadian regulation, consequently leading to or increasing the magnitude of sleep and circadian alterations (Wang et al., 2015). Conversely, sleep and circadian alterations affect several processes such as glymphatic clearance, brain metabolism, oxidative stress, and neuroinflammation, all of which could be involved in the physiopathology of neurodegenerative diseases (Leng et al., 2019). In this regard, findings by Kong et al. provided insight into the possible mechanisms by which obstructive sleep apnea (OSA) could be associated with cognitive decline. These authors demonstrated that moderate to severe OSA patients (apnea-hypopnea index ≥15) exhibited disturbed functional connectivity from the dorsal anterior insula, an important area involved in cognitive processing, to several areas relevant to cognition. The report presented by Kazukauskiene et al. demonstrated that an interaction between OSA and high levels of N-terminal pro-B-type natriuretic peptide in males with coronary artery disease increases the risk of cognitive functioning problems. Furthermore, Needham et al. proposed a dichotomous role for brain-type fatty acid binding protein (FABP7) in the sleep-mediated pathogenesis of AD. The intracellular lipid chaperone FABP7 regulates fatty acid uptake and transport, being implicated in sleep regulation and neurodegenerative diseases, but the mechanisms underlying this are unclear. According to these authors' hypothesis, depending on the availability of arachidonic acid (AA) and docosahexaenoic acid (DHA), FABP7 could bind to either AA, leading to a cascade of events promoting wakefulness aligned with AD pathogenesis, or to DHA with beneficial effects including activation of anti-inflammatory pathways and neuroprotection.

Interventional studies aid in the unraveling of possible relationships of causality between sleep, circadian rhythms, and neurodegenerative diseases. Giménez et al. investigated the feasibility and long-term continuous positive airway pressure (CPAP) compliance in Down Syndrome patients with OSA. The findings demonstrated an appropriate tolerance to CPAP treatment among these patients, indicating the suitability of this population for future clinical trials aiming to evaluate the preventive role of sleep in the context of AD.

In summary, the studies presented in this Research Topic reinforce a bidirectional relationship between sleep and circadian alterations and neurodegenerative diseases. Nevertheless, future evaluations are required to address the several gaps and limitations within the current knowledge. To accomplish this, the consideration of the following items would be of great value: (i) inclusion of appropriate assessments of sleep and circadian health using validated questionnaires and objective measurements; (ii) incorporation of circadian function markers other than the rest-activity rhythm to increase reliability of the findings and dissociate the effects related to sleep to those related to the circadian rhythms; (iii) interpretation of findings based on pharmacological treatment, mental health disorders, disorientation, decreased exposure to natural light, loss of autonomy, and dependence of caregivers schedules; (iv) incorporation of molecular markers to evaluate the impact of sleep and circadian health on the physiopathology of the disease besides the clinical manifestations; (v) performance of experimental and interventional studies to further understand the possible mechanisms and relationships of causality within this context. Accordingly, while there is sufficient evidence related to the impact of neurodegenerative diseases on sleep, the evidence in the reverse direction is mainly based on association and correlational studies. These would collectively contribute to improving the knowledge in this regard, ultimately enlightening promising strategies to prevent or delay the progression of neurodegenerative diseases.

## Author contributions

All authors listed have made a substantial, direct, and intellectual contribution to the work and approved it for publication.
